# Expert teacher based on foundation image segmentation model for object detection in aerial images

**DOI:** 10.1038/s41598-023-49448-9

**Published:** 2023-12-11

**Authors:** Yinhui Yu, Xu Sun, Qing Cheng

**Affiliations:** School of Communication Engineering, Jilin University, Changchun, 130012 Jilin China

**Keywords:** Computer science, Information technology, Electrical and electronic engineering, Imaging techniques, Imaging and sensing, Aerospace engineering

## Abstract

Despite the remarkable progress of general object detection, the lack of labeled aerial images limits the robustness and generalization of the detector. Teacher–student learning is a feasible solution on natural image domain, but few works focus on unlabeled aerial images. Inspired by foundation models with the powerful generalization in computer vision field, we propose an expert teacher framework based on foundation image segmentation model called ET-FSM. Our approach provides the performance gains for the student detector by generating high-quality pseudo-labels for unlabeled aerial images. In the ET-FSM, we design the binary detector with expert guidance mechanism to sufficiently leverage the extra knowledge obtained from the foundation image segmentation model, which accurately detects object positions in the complex backgrounds. Also, we present the momentum contrast classification module to distinguish confused object categories in aerial images. To demonstrate the effectiveness of the proposed method, we construct an unlabeled aerial image dataset covering various scenes. The experiments are conducted on diverse types of student detectors. The results show that the proposed approach achieves superior performance compared to existing methods, and allows the student detector to achieve fully supervised performance with much less labeled aerial images. Our dataset and code are available at https://github.com/cq100/ET-FSM.

## Introduction

Object detection for aerial images captured by UAVs (unmanned aerial vehicles) has been widely used in numerous practical applications, such as traffic surveillance, disaster relief and smart agriculture^1,2^. Although general object detection has made prominent success since the rise of deep learning, the complex working environment of UAVs and the scarcity of labeled aerial images impair the robustness and generalization of the detector, which limits the advancement and application of aerial image detection^3,4^. Current works mainly focus on data augmentation and elaborate network architecture design to improve the detector performance^5,6^. Nevertheless, these methods ignore the potential application of unlabeled aerial images available everywhere^7^.

Recent semi-supervised object detection in natural images has obtained performance gains from a large number of unlabeled images by leveraging the teacher-student learning manner^8,9^. Typically, this methodology adopts a complex and high-performance teacher model to generate pseudo-labels for unlabeled images, and then these pseudo-labels and ground-truth labels are used to train a lightweight student model^10^. In the training process, accurate pseudo-labels are critical that can provide the correct supervision information to the student model^11,12^. Nevertheless, aerial images usually contain small objects and complex backgrounds, which makes teacher models generate inaccurate predictions^13^.

Large language foundation models can generalize to unseen data distributions by training with abundant text corpora, such as GPT-4^14^. Inspired by this, the foundation models in computer vision field are also developing rapidly^15,16^. The segment anything model (SAM) released by Meta AI Research is the most representative in semantic segmentation tasks^17^. The model is trained on over one billion masks, and constructs a data collection loop to continuously enhance zero-shot and few-shot generalization. The unique property can assist the detector to resist noise disturbances in aerial image detection tasks.

To this end, we design an effective teacher framework based on foundation image segmentation model for object detection in aerial images. The proposed approach can transfer knowledge learned from unlabeled aerial images to the student detector, which makes the student detector achieve superior performance with a small number of labeled aerial images. Specifically, we propose a binary detector with expert guidance mechanism (EGD) to achieve the finer bounding box prediction by incorporating the guidance information provided from the foundation image segmentation model. Also, the momentum contrast classification (MCC) module is designed for object classification, which is able to distinguish confused object categories and boost the feature representation ability. The two key components can be used to generate accurate pseudo-labels for objects in complex aerial images. To prove the validity of the proposed method, we collect 14110 unlabeled aerial images under different scenes and conduct extensive experiments on Visdrone^18^ and UAVDT^19^ datasets. Our dataset and code are available at https://github.com/cq100/ET-FSM.

The primary contributions of our paper are as follows:

- We present an expert teacher framework based on foundation image segmentation model called ET-FSM, which uses the unlabeled aerial images with high-quality pseudo-labels to enhance the robustness and generalization of the student detector. We also construct an unlabeled aerial image dataset to provide valuable resources for unlabeled data study in aerial image detection.
- We design the binary detector with expert guidance mechanism (EGD) that treats the extra knowledge provided from the foundation image segmentation model as new image modality information. It is able to resist the background disturbances and accurately locate object positions.
- We propose the momentum contrast classification (MCC) module. It regards object features of the same class as a cluster, and uses the cluster expectations for object classification, producing higher-quality pseudo category labels for easily confused objects in the aerial images.

## Related work

### Object detection in aerial images

Different from natural images, UAVs usually capture aerial images under varying illumination and uncontrolled outdoor conditions, which requires object detection models with strong robustness^20,21^. Existing methods are mainly carried out in terms of model structure and labeled data^22^.

Designing elaborate model architectures facilitates better extraction of small object features. Nuisance disentangled feature transform^23^ designed the extra nuisance prediction branch to learn robust features for each domain covering altitude, view and weather. Cascaded zoom-in detector^24^ was a recent method that reused detectors based on object density in the training and inference stages. This manner brings tremendous computational costs, which is difficult to deploy on embedded UAV platform.

Using large-scale labeled aerial image dataset to train detectors can intuitively enhance robustness, but manually labeling objects is time-consuming and labor-intensive^25^. Data augmentation expands the dataset by providing a diverse view of the sample. Uniform cropping^26^ as a popular augmentation approach divided the aerial image into four equal-sized patches, and then merged these patches into the training set. Mask Re-sampling^27^ generated numerous object chips form dataset, and used masks to determine proper positions for these chips. These methods still extract features from the labeled aerial image dataset without utilizing unlabeled aerial images easily available.

### Semi-supervised object detection

In recent years, semi-supervised learning has gradually focused on object detection tasks, which can be divided into consistency-based approach and pseudo labeling approach^28,29^. The latter is a mainstream method that leverages the teacher model trained on ground-truth labels to generate pseudo-labels for unlabeled images, and retrains the student model with all data.

STAC^30^ first followed the popular teacher-student learning framework to achieve semi-supervised object detection. Unbiased Teacher^31^ utilized the threshold to select more reliable pseudo-labels for student models. Soft Teacher^32^ dynamically adjusted the training loss weights for each pseudo-box, which alleviated the negative effects of incorrect pseudo-labels. Despite significant progress in natural images, these teacher models cannot be directly applied to aerial images. The bounding boxes of small objects in aerial images are particularly sensitive to noise perturbations, which lead to the unreliable prediction from the teacher model. ScaleKD^33^ was the recently released teacher-student learning scheme specifically for small object detection, and it designed a cross-scale assistant to reduce the adverse effect of teacher model. ZoomInNet^34^ distilled a standard teacher model by learning cross-scale knowledge of small objects.

## Proposed method

### ET-FSM overall

**Figure 1:**
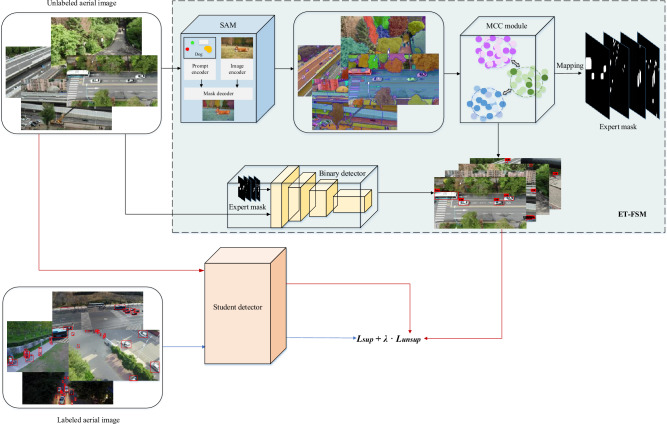
The overall architecture of the proposed method based on SAM.

When detecting small and confused objects in aerial images, general teacher detectors have poor performance against complex background disturbances. To generate more accurate object bounding boxes and category soft labels, we decouple object localization and classification tasks. The overall architecture of the proposed approach based on SAM is presented in Fig. 1. Firstly, we employ the SAM to segment all objects in the aerial image. These segmented regions are categorized and mapped into expert masks. Afterwards, the expert mask serves as an image modality to guide the binary detector to predict more accurate object positions. Finally, the MCC module determines the specific category scores of these detected objects.

The ET-FSM is responsible for pseudo-label generation, and the student detector is optimized with these pseudo-labels and ground-truth labels. In principle, the student detector is arbitrary. We use Faster R-CNN^35^ as a baseline example. The optimization loss *L* for student detector is calculated as follows:

1 $$ L = L_{{sup}}  + \lambda  \cdot L_{{unsup}}  $$

where $L_{sup}$ and $L_{unsup}$ are the loss functions computed on the labeled aerial images and unlabeled aerial images respectively, and $\lambda$ is a hyperparameter to balance the two losses.

2 $$\begin{aligned} \begin{aligned} L_{sup}&=L_{cls}+L_{reg} \\&=\sum _i\left( {\text {CE}}\left( x_{cls}^i, y_{cls}^i\right) +{\text {Smooth}}_{\textrm{L} 1}\left( x_{reg}^i, y_{reg}^i\right) \right) \end{aligned} \end{aligned}$$

where $L_{cls}$ is the classification loss, $L_{reg}$ is the regression loss, CE is the cross-entropy loss function, $x_{i}$ is the predicted sample, and $y_{i}$ is the ground-truth label.

The $L_{unsup}$ loss is similar to the $L_{sup}$ loss. The difference is that we employ the pseudo category soft labels generated by the MCC module for classification, and pseudo location labels obtained by the EGD for regression.

### Aerial image dataset collection

To explore the performance gains of the proposed ET-FSM on the student detector, we construct an unlabeled aerial image dataset containing 14110 visible frames. This available resource is exceptionally valuable in studying unlabeled data and enhancing the detection capabilities of aerial images.

**Figure 2:**
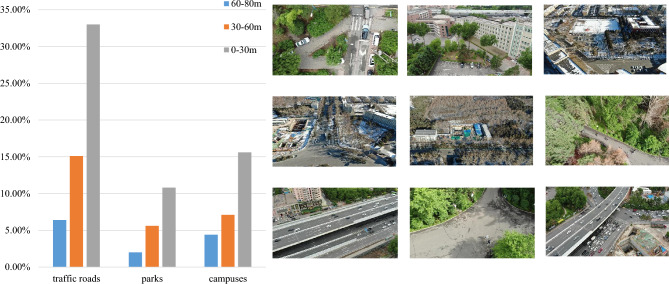
Distribution of images across capturing scenes and heights.

**Figure 3:**
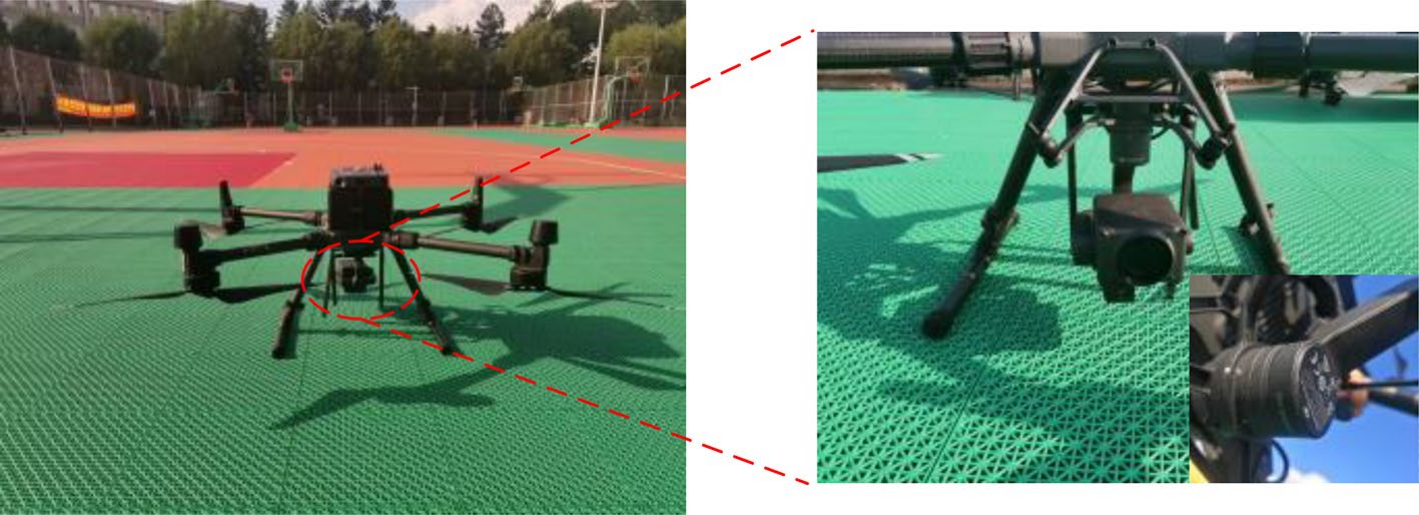
The hardware devices.

Our dataset covers multiple scenes including traffic roads, campuses and parks. The resolution of collected image is 1920$\times$1080. To increase the adaptability of high-altitude missions, these images are captured in different height intervals of 0-30m, 30-60m, and 60-80m. We provide the statistical information of different shooting scenes and heights in Fig. 2. For hardware devices, we select the DJI Matrice M300 RTK UAV equipped with the Zenmuse H20 sensor to collect data, as shown in Fig. 3. The relevant equipment parameters are shown in Table 1.

**Table 1:** Relevant equipment parameters.

Devices	Parameters	Values
DJI Matrice M300 RTK	Max payload	2.7 kg
	Hovering accuracy	Vertical:±0.1m
		Horizontal: ±0.3m
	Max angular velocity	Pitch: 300$^{\circ }$/s
		Yaw: 100$^{\circ }$/s
Zenmuse H20	Sensor	1/2.3” CMOS
	Focal length	4.5mm
	Aperture	f/2.8

### Binary detector with expert guidance mechanism

The SAM is a foundation image segmentation model, and has the powerful zero-shot generalization ability. When directly applying to aerial images, the segmentation performance of SAM may be unsatisfactory due to sensitivity to environmental perturbations^36^. Inspired by the multimodal object detection approach in aerial images^37^, we consider the extra knowledge from SAM as new modality information to help the detector focus on the relevant object regions. Specifically, we propose a binary detector with expert guidance mechanism (EGD) to generate trustworthy bounding boxes for unlabeled aerial images.

**Figure 4:**
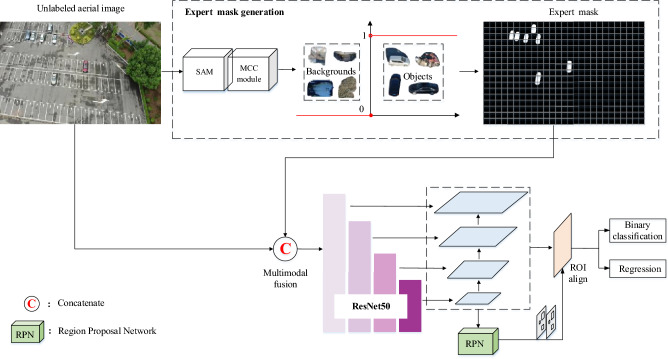
Binary detector with expert guidance mechanism.

The workflow of the designed detector is shown in Fig. 4. In the training stage, we employ the SAM to segment the labeled aerial images into multiple regions, and use the MCC module to distinguish the objects and backgrounds in the segmentation results. The pixel values of object regions are set to 1 and the other regions are set to 0. The corresponding expert mask is generated, and stored in the local environment. Different epochs avoid duplicate segmentation operations, which greatly saves computational complexity and time costs.

In the inference stage, the unlabeled aerial images are employed to generate expert masks. The expert mask concatenates with the original image along the channel dimension as the binary detector input to provide the guidance information. Moreover, our detector only performs binary classification of objects and backgrounds for accurate object location. We adopt the Faster R-CNN as the binary detector in this paper.

### Momentum contrast classification module

Since the disturbances caused by flying altitude, viewing angle, and weather condition are more severe in aerial images, objects of different categories usually have the similar appearances. It is difficult for a general classifier to distinguish multiple confused object categories. Inspired by the momentum contrast learning^38^, we propose the momentum contrast classification (MCC) module to generate the accurate pseudo category labels. Our module can be combined with most image classifiers. We use the PVTv2 (pyramid vision transformer version2)^39^ in this paper.

The MCC module uses the classifier to encode input samples. Sample features of the same class are regarded as a cluster. The expectation vectors of the clusters are used for contrast classification. By minimizing the contrast classification loss $L_c$, our module increases the similarity of object features from the same category, and dissimilarity to that of different categories.

3 $$\begin{aligned} L_c=-\log \frac{\exp \left( q \cdot c_{+} / \tau \right) }{\sum _{i=0}^C \exp \left( q \cdot c_i / \tau \right) } \end{aligned}$$

where *q* is an input encoded sample vector, $c_{+}$ is the expectation of encoded vector for the matched category, $c_{i}$ is that of the category *i*, and $\tau$ is a temperature parameter. Each input encoded sample vector that completes the calculation is stored in a queue. When the maximum value *Q* of the queue capacity is reached, the encoded sample vectors for each category are updated through momentum.

4 $$\begin{aligned} c_i \leftarrow c_i+(1-m) c_i^{\prime } \end{aligned}$$

where *m* is a momentum parameter, and $c_i^{\prime }$ is the expectation of updated encoded vectors for category *i*.

**Figure 5:**
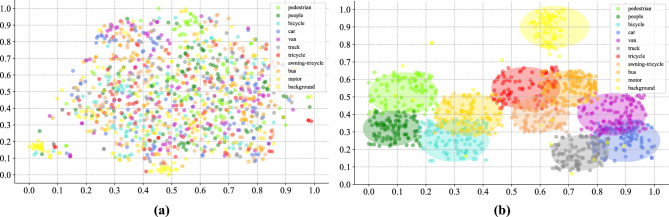
Encoded feature distribution (**a**) without the MCC module (**b**) with the MCC module.

In the training stage, we use the segmented background and object regions from the labeled aerial images. In the inference stage, the MCC module distinguishes objects and backgrounds output from the SAM on the unlabeled aerial images, and determines the specific category scores of objects output from the binary detector. Figure 5 presents encoded feature distribution. It can be observed that high-dimensional features from different categories lack clear representation of distribution boundaries in the dimensionality reduction visualization. After applying the MCC module, the feature points of the same category are more clustered. This indicates that the MCC module is conducive to distinguishing between easily confused objects from different categories.

## Experiments

### Implementation detail

The experiments are conducted on the Visdrone^18^ and UAVDT^19^ datasets, which provide 10209 images and 38327 images with annotations, respectively. We measure the performance gains of the proposed method on the student detector by adding the aerial images with pseudo-labels. To further test robustness, we introduce ten corrupted types for the two testing sets to simulate the UAV-specific perturbations, which is the same setting as in the previous method^40^.

Our method is trained on a NVIDIA Tesla P40 GPU platform with 24GB memory, and the implementation is based on the MMDetection toolbox^41^. The input image size of student detectors is set to 1000 $\times$ 600 pixels. We set the batch size to 4, the initial learning rate to $1.0 \times 10^{-4}$ and the epochs to 18. For the evaluation metric, we mainly adopt the AP (average precision), AP50, and AP75 to measure the detection performance.

### Comparison experiments

We employ three types of base student detectors based on ResNet50^42^ to evaluate the performance gains of our method, including anchor-based Faster R-CNN (FRCNN)^35^, RetinaNet^43^ (Retina), and anchor-free FCOS^44^. Also, we use the advanced standard-scale detector DINO^45^ and the recently released UAV-specific detector CEASC^46^ as student models. Table 2 shows the detection results of different student detectors. As can be seen, the ET-FSM improves all detector performance, validating the effectiveness of our approach. In particular, after using the proposed approach, the base detectors are able to achieve competitive performance with the advanced standard-scale DINO and UAV-specific CEASC. The accuracy improvement on the Visdrone dataset is greater than on the UAVDT dataset. One reasonable explanation is that the UAVDT contains more labeled aerial images and fewer categories, which lowers the top bound on performance growth. Moreover, it can be seen that the AP75 score increase is smaller than the AP50 score, because there exist some position deviations when generating pseudo-labels. After adding the UAV-specific perturbations, our approach increases the AP scores of the base student detector by 6.6$\%$, 7.0$\%$, and 7.6$\%$ on the corrupted Visdrone dataset, respectively. These results indicate that the proposed method has considerable corruption robustness gains.

**Table 2:** The detection results of different student detectors.

Method	Visdrone						UAVDT					
	Clean accuracy			Corruption robustness			Clean accuracy			Corruption robustness		
	AP (%)	AP50 (%)	AP75 (%)	AP(%)	AP50 (%)	AP75 (%)	AP (%)	AP50 (%)	AP75 (%)	AP (%)	AP50(%)	AP75 (%)
FRCNN	21.4	37.4	21.5	16.6	20.9	16.3	17.1	29.2	18.6	10.4	18.9	9.2
Retina	15.7	29.8	14.6	11.1	23.6	10.2	15.8	30.2	15.3	12.8	24.8	11.7
FCOS	16.9	29.8	17.2	9.8	19.2	9.3	16.9	29.9	17.8	10.5	19.8	10.0
DINO	23.1	41.8	22.0	19.7	36.8	18.1	16.2	28.8	16.8	14.8	26.7	14.9
CEASC	19.5	32.1	20.4	14.9	25.9	15.3	16.7	27.3	19.0	14.9	25.2	16.3
FRCNN+ET-FSM	25.8	45.9	23.4	23.2	39.8	18.8	19.4	32.6	19.2	13.9	24.8	10.5
Retina+ET-FSM	21.1	39.3	17.7	18.1	29.8	14.3	17.2	32.5	15.6	15.2	24.9	11.9
FCOS+ET-FSM	20.6	34.4	18.6	17.4	28.1	12.7	18.1	32.5	18.0	12.2	24.7	10.3
DINO+ET-FSM	26.3	52.9	24.0	24.1	45.5	20.4	18.9	32.0	20.5	17.7	29.5	19.3
CEASC+ET-FSM	22.7	41.3	21.5	18.9	34.1	18.6	18.5	31.7	19.8	16.7	26.5	19.4

Table 3 provides the AP scores for each category on the clean Visdrone dataset. We compare the performance of the student model using ground-truth labels, adding the pseudo-labels generated by the student model itself (SMI), and the pseudo-labels generated by the proposed ET-FSM. It can be observed that directly utilizing the student model to generate pseudo-labels deteriorates the detection performance. We assume the reason for this phenomenon is that the student model predicts pseudo-labels with imprecise object bounding boxes and severe category confusion, leading to error accumulation. The ET-FSM method not only largely boosts the detection accuracy of FRCNN on categories with most training instances, but also improves AP scores on the long tail categories.

**Table 3:** The AP scores for each category on the clean Visdrone dataset.

Method	AP (%)	AP50 (%)	AP75 (%)	Car	Bus	Van	Ped.	Motor	Truck	Person	Tricycle	Awn.	Bicycle
FRCNN	21.4	37.4	21.5	51.2	32.2	27.8	20.4	20.1	20.1	13.7	13.5	7.7	7.0
FRCNN+SMI	15.8	29.3	15.0	46.5	17.1	21.8	16.3	14.9	14.7	10.7	8.2	4.1	3.7
FRCNN+ET-FSM	25.8	45.9	23.4	64.8	40.1	34.0	24.8	24.0	24.0	16.0	14.8	7.5	7.7

We compare the performance of existing approaches on the clean Visdrone dataset in Table 4. For a fair comparison, we use ResNet50 as the backbone network, and implement these models under the same experimental conditions. In the inference stage, we do not perform any cropping operation. Indeed, the uniform cropping^26^ and the cascaded zoom-in detector^24^ increase the AP scores, but the improvement is marginal. Compared to the two methods, our approach achieves better performance. It suggests that sufficiently leveraging unlabeled aerial images through the proposed teacher framework does bring in greater gains. We also compare the advanced Soft Teacher^32^ in the field of natural images, and ZoomInNet^34^ and ScaleKD^33^ in the field of aerial images, which are based on the teacher-student learning framework typically used for semi-supervised methods. It can be observed that the ET-FSM outperforms these comparative methods, and achieves higher AP score increases of 4.4$\%$ and 5.4$\%$ on the FRCNN and Retina, respectively. This means that our method can effectively boost the small object detection ability of the student detector.

**Table 4:** Performance comparison of existing approaches on the clean Visdrone dataset.

Method	AP (%)	AP50 (%)	AP75 (%)
FRCNN	21.4	37.4	21.5
Uniform cropping^26^	22.7	40.7	22.2
Cascaded zoom-in^24^	23.9	42.2	24.0
Soft Teacher^32^	24.5	39.3	27.0
ET-FSM	25.8	45.9	23.4
Retina	15.7	29.8	14.6
ZoomInNet^34^	17.3	33.3	16.3
ScaleKD^33^	19.4	36.8	18.0
ET-FSM	21.1	39.3	17.7

### Ablation study

**Figure 6:**
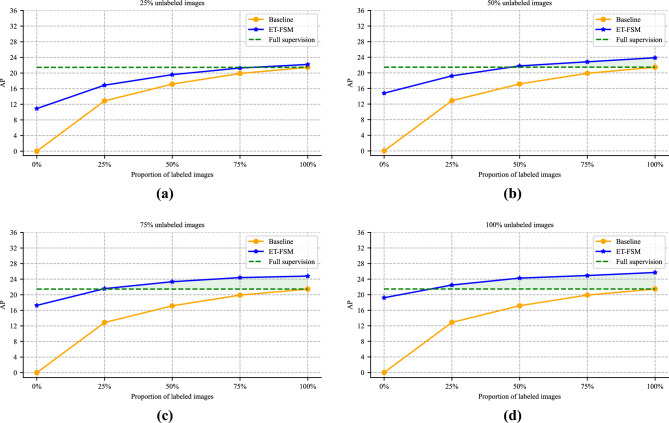
The AP scores comparison of FRCNN baseline and the ET-FSM under varying proportions of unlabeled images.

Our ablation experiments are conducted on the clean Visdrone dataset. We evaluate the AP scores of FRCNN baseline and the ET-FSM on different unlabeled image proportions in Fig. 6. It can be observed that our approach can achieve greater performance gains with fewer labeled samples. When adding 75$\%$ unlabeled images, the ET-FSM can surpass 100$\%$ fully supervised performance only using 25$\%$ labeled data.

We further investigate the effect of the designed momentum contrast classification (MCC) module on the classification performance in Table 5. As can be seen, using the MCC module can increase the macro-average score by 13.6$\%$ compared to vanilla classifier, demonstrating the effectiveness of our module. Also, we explore the value *Q* of the queue capacity. When the *Q* is 512, the highest macro-average score of 85.4$\%$ and micro-average score of 93.5$\%$ can be obtained.

**Table 5:** The effect of each component in the ET-FSM classification.

Vanilla classifier	MCC module	*Q*=128	*Q*=256	*Q*=512	*Q*=1024	Macro-average	Micro-average
$\checkmark$						71.8	84.3
	$\checkmark$	$\checkmark$				81.3	89.8
	$\checkmark$		$\checkmark$			82.1	90.6
	$\checkmark$			$\checkmark$		85.4	93.5
	$\checkmark$				$\checkmark$	83.5	93.8

Table 6 shows the ablation study of the binary detector with expert guidance mechanism (EGD). In the ET-FSM, using the expert mask to train the detector has higher accuracy than directly inputting the original aerial image. For example, the AP scores increase by 14.8$\%$ and 13.7$\%$ on the Visdrone and UAVDT datasets, respectively. The results show that our detector can achieve more accurate object bounding box prediction.

**Table 6:** The ablation study of the EGD.

Method	Visdrone			UAVDT		
	AP (%)	AP50 (%)	AP75 (%)	AP (%)	AP50 (%)	AP75 (%)
Vanilla detector	37.1	65.7	37.1	36.2	64.1	37.8
EGD	51.9	91.0	51.7	49.9	87.7	49.8

**Figure 7:**
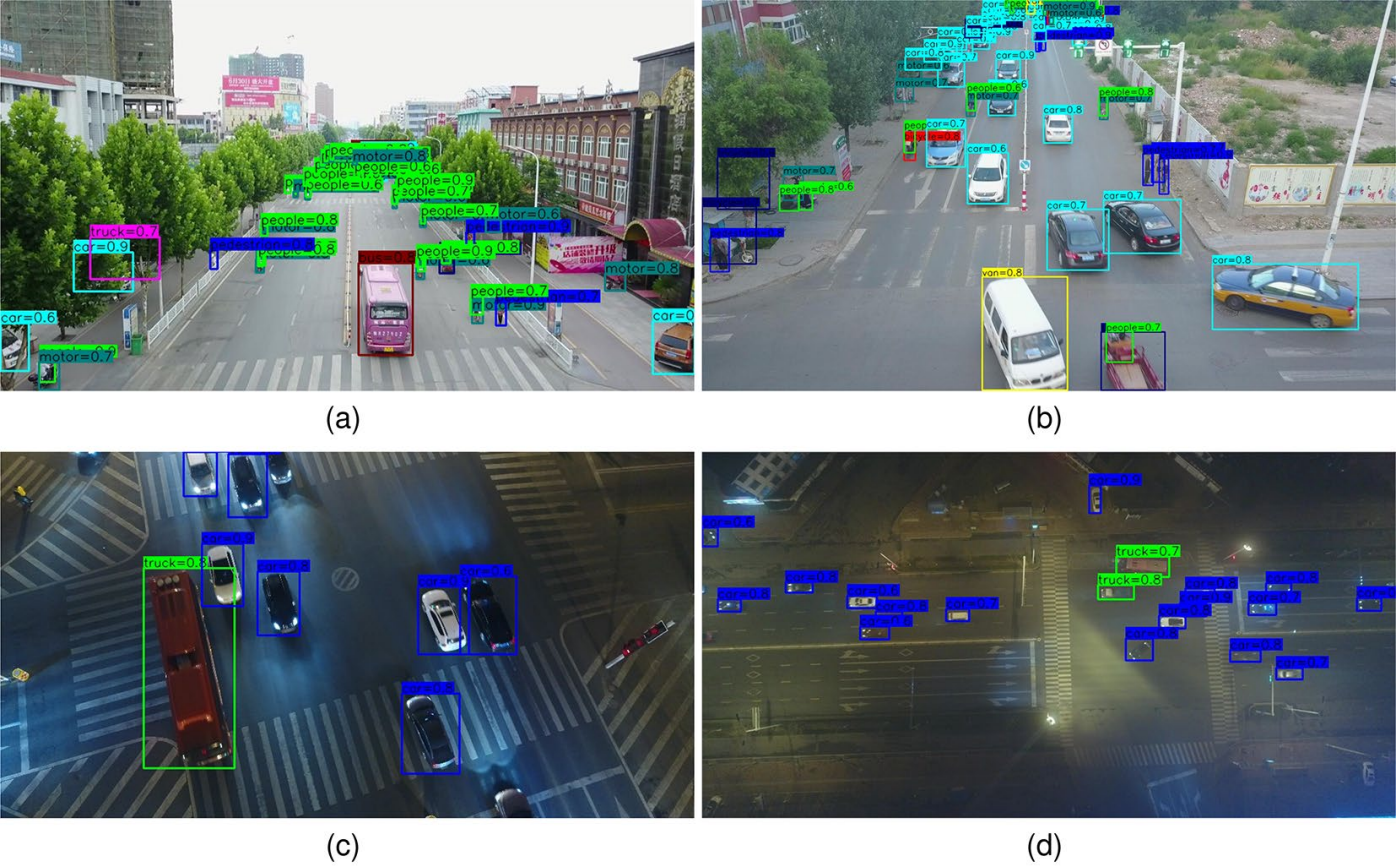
The visualizing detection results of our approach on the Visdrone and UAVDT datasets.

Figure 7 shows the visualizing detection results of our approach on the Visdrone and UAVDT datasets. It can be seen that small and occluded objects can be detected by using the ET-FSM, and their categories can be clearly identified, such as motor and people. In particular, the proposed method is able to detect the confused objects with the complex background in the poorly illuminated scenes.

## Conclusion

We propose an expert teacher framework ET-FSM based on foundation image segmentation model to boost the robustness and generalization of student detectors in aerial images. Our approach takes full advantage of the effective knowledge from the powerful foundation image segmentation model to generate accurate pseudo-labels for unlabeled aerial images. Specifically, we design the binary detector with expert guidance mechanism (EGD) and the momentum contrast classification (MCC) module in the ET-FSM to make teacher models predict more accurate location bounding boxes and object category scores. Moreover, we collect an unlabeled aerial image dataset in various real-world scenes, which provides abundant resources for unlabeled aerial image research. The experiment results show that the proposed method brings greater performance gains than advanced methods, and enables the student detector to outperform 100$\%$ supervised performance with only 25$\%$ labeled images when adding 75$\%$ unlabeled images.

## Data Availability

The dataset and code of the current study are available in the github repository, https://github.com/cq100/ET-FSM.
